# Reducing the burden of psychological questionnaire measures through selective item re-weighting

**DOI:** 10.1098/rsos.241857

**Published:** 2025-04-16

**Authors:** Toby Wise, Nura Sidarus

**Affiliations:** ^1^ Department of Neuroimaging, King’s College London, London, UK; ^2^ Department of Psychology, Royal Holloway University of London, Egham, UK

**Keywords:** measure development, questionnaires, psychometrics

## Abstract

Questionnaire measures are central to many areas of study within the psychological sciences. However, they often place a heavy burden on participants; questionnaires are frequently lengthy and unengaging, and with participants often required to complete multiple measures within a single study, this results in lower data quality, increased cost and a poor participant experience. Here, we introduce a straightforward method for creating short versions of existing measures that are able to accurately determine participants’ sum scores, subscale scores or factor scores. Our method, referred to as Factor Score Item Reduction with Lasso Estimator, uses Lasso-regularized regression to select items and weight them such that true scores can be predicted accurately from a reduced item set. We demonstrate the performance of this method on an example dataset, and provide code and guidance for implementing the approach.

## 1. Introduction

Self-report questionnaire measures are central to much psychological research, providing a practical means of assessing psychological constructs and capturing individual differences. Such measures are typically validated extensively, ensuring that they provide robust, valid and reliable measures of a particular construct.

However, self-report measures can often be lengthy and repetitive, with large numbers of items addressing similar constructs, and become time-consuming and monotonous as a result. This places a significant attentional burden on participants and can lead to disengagement and poor-quality responses [1,2]. Furthermore, participants often find lengthy studies to be off-putting when considering participation in research [3,4]. Finally, time-consuming measures inevitably result in longer participation times and hence greater spending on participant payments, limiting available funding and restricting sample sizes.

Prior efforts to reduce the burden of self-report measures by constructing short scales have taken a variety of approaches [5]. Perhaps the most effective and commonly used approach uses item response theory [6,7], in which models are constructed that describe the relationship between an individual’s response to a given item and their score on the underlying construct. These models can be used to select items whose responses are most discriminative in relation to the underlying construct being measured, resulting in short measures that accurately measure the construct of interest [8–11].

While this approach can be effective, it does possess certain limitations. Item response theory requires proper specification of the item characteristic curve model, for which there is not necessarily an optimal approach [12]. Parameters of these models must also be estimated accurately, which presents additional challenges [13], and it is important to ensure that model fit is acceptable before drawing inferences regarding the value of individual items [14]. More significantly, these models typically assume that the measure is unidimensional, having only a single underlying dimension [6]. Violations of this assumption can invalidate model parameters [15], rendering the approach infeasible for measures that assess multiple latent constructs. This is an important limitation, as many measures are multidimensional, as is often demonstrated through factor analytic approaches revealing multiple underlying latent dimensions. This is often the case in scales designed to measure symptoms of mental health problems, which will frequently assess multiple sub-dimensions of a more general symptom. In sum, while effective, item response theory approaches are complex and require expertise, while also being limited to unidimensional measures.

The need for shorter scales is not limited to the case of individual measures targeting a specific latent construct. Increasingly, researchers are looking to factor analysis to identify broader latent dimensions captured by existing measures of related constructs. For example, in mental health research, we may wish to identify transdiagnostic symptom dimensions that can be captured by combining multiple measures of specific symptoms and performing factor analysis [16,17]. Given the burden placed on participants by completing multiple scales, resulting in hundreds of items, this is another area where it is desirable to derive a shorter scale that can nonetheless capture these latent dimensions. We have previously used an earlier variant of the approach presented here in this context successfully [18,19].

Here, we introduce a simple, data-driven approach for reducing the length of self-report questionnaire measures which is straightforward to use, does not require fitting of complex models, and can be applied to multidimensional measures (i.e. scales measuring multiple latent constructs, potentially with established subscales, or combinations of existing scales). This approach, which we refer to as factor score item reduction with Lasso estimation (FACSIMILE), derives a linear weighted combination of items that accurately predicts scores derived from the full-length measure, providing a straightforward way to derive brief item sets automatically.

## 2. Material and methods

### 2.1. The FACSIMILE method

Here, we introduce the principle behind the FACSIMILE method. We assume that observed scores (y) derived from a questionnaire measure (these may be total sum scores, subscale sum scores or factor scores derived from factor analysis) can be approximated ($\hat{y}$) subject to some degree of error (ϵ) as a linear weighted sum of individual item scores ($x_{1},x_{2},x_{3},\ldots )$:


(2.1) $$\begin{array}{c} \hat{y}=w_{1}\times x_{1}+w_{2}\times x_{2}+w_{3}\times x_{3}+\cdots +\epsilon ,\;\; \end{array}$$

where $w_{n}$ represents the weight of item $x_{n}$. With the full item set, this is a straightforward and perfectly accurate prediction (i.e. the error term ϵ is zero); if predicting sum scores, all weights $w_{n}$ are 1, while if using factor scores, the weights correspond to the weights derived from factor analysis. However, when we remove items, this becomes an imperfect prediction due to the loss of necessary items, hence the inclusion of the error term ϵ.

This represents a standard linear regression model, and we can therefore apply existing techniques to identify which predictors (in this case, which items $x_{n}$) are most predictive of our target variable (in this case, the observed sum score y). Given that our aim is to drop items that are less informative of the observed sum score, we turn to the Lasso (also referred to as L1) estimator, which in effect removes uninformative predictors by setting their weight ($w_{n}$) to zero. The remaining items are reweighted to ensure that an accurate prediction is maintained. We can estimate these weights using standard optimization procedures as implemented in commonly used software packages (e.g. scikit-learn for Python).

This provides a subset of items that can be used to accurately predict the observed scores when weighted appropriately. For example,


(2.2) $$\begin{array}{c} \hat{y}=w_{1}\times x_{1}+0\times x_{2}+w_{3}\times x_{3}+\ldots +\epsilon .\;\; \end{array}$$

Here, the weight of item [2] has been set to zero, meaning it has effectively been dropped from the measure. The weights of the remaining items ($w_{1}$ and $w_{3}$) will have changed to ensure that the observed score is still predicted accurately.

A helpful feature of the Lasso approach is that it provides a hyperparameter (α) that can be used to determine how selective the algorithm is in setting item weights to zero: values close to zero will include more items, whereas higher values will be more selective. Thus, we can adjust this parameter to determine the number of included items, and accordingly how brief a revised measure will be. This will, in turn, affect the accuracy of the measure, since removing items will unavoidably impact upon the accuracy of predictions.

Importantly, there is not necessarily a correct or optimal value of α; some applications may accept a very brief measure at the expense of accuracy, whereas others may prefer a slightly shortened measure that retains high accuracy. As such, we cannot recommend any particular value of the parameter, but this should instead be chosen based on (i) the original number of items in the measure, since the value of α will depend on how many items are present initially and (ii) how short a scale the researcher wishes to create. Typically, it may be simplest to evaluate candidate values of α through trial-and-error, but the ideal method for finding the best value of α is through a relatively exhaustive grid search procedure (described in the following section).

This represents a simple problem in the case of a unidimensional measure, where Lasso-regularized regression can be directly applied to select a subset of items. However, this becomes more complex for multidimensional measures (e.g. scales with multiple subscales, or when estimating latent multiple latent factors). In testing, we found that the best performance is typically not achieved with a consistent value of α across dimensions; rather, each dimension (i.e. a factor or questionnaire subscale) is typically best predicted by a model with a different value of α. As a result, we are not able to use a typical multi-task Lasso regression model [20,21] that assumes a single value of α for each dimension. Instead, we work around this limitation by using a two-step procedure. First, we select items for each dimension independently using the Lasso estimator described above, providing a set of included items with relevance for each dimension. This enables the procedure to be more or less restrictive in its inclusion threshold depending on the requirements of each dimension; if a given dimension is straightforwardly estimated based on a few included items, then a high α value will suffice, whereas more challenging dimensions to predict will require lower values, and hence more items included.

Second, we restrict our dataset to those items included in any one of these models (i.e. items retained when predicting at least one of the dimensions) and fit individual unregularized regression models predicting each of the target dimensions from the included items. This ensures that we utilize information present in all of the included items for predicting every dimension, even if the initial variable selection step did not suggest the inclusion of a given item for a particular dimension. For example, if one dimension requires a larger number of items to be included, we ensure that we also use these for enhancing the predictions of other dimensions, even if they provide relatively little added value. As mentioned previously, this second step is redundant for unidimensional measures. These two steps are integrated into a single function in the provided Python package, and therefore do not necessarily need to be implemented directly.

### 2.2. Evaluation

The accuracy of predicted scores can be determined according to any established metric for continuous predictions; we use $R^{2}$ as it provides a simple and intuitive measure of accuracy. As with any prediction task, it is important to evaluate performance on a dataset that is independent of that on which the model was trained. As such, we divide our data into three subsets: training, validation and testing. The training set is used for training the model (i.e. deriving the weights on each item); the validation set is used for evaluating the performance of the model according to the value of hyperparameter α; the testing set is used for evaluating the performance of the final model.

In practice, the simplest method for identifying the best model is to use a procedure that tests various values of α within a given range, providing an indication of how performance (and the number of items included) vary according to the value of this parameter. The results of this procedure can then provide candidate short versions of the initial measure with varying lengths and predictive accuracy. We use a randomized search procedure [22], drawing possible values of α from a beta distribution Beta(1,3), as this over-samples lower values of α that are more likely to be effective in reducing the number of items. The value of α is dependent upon the number of items in the original measure, and so this distribution can be adapted accordingly to ensure that the values used are appropriate. The number of iterations required will depend on the complexity of the question, and is most dependent upon the number of target variables being estimated (e.g. the number of subscales). For the examples reported here, we use 1000 iterations.

As mentioned above, there is no correct value of the α parameter. Nevertheless, we can attempt to find a value of α that provides a generally acceptable balance between brevity and accuracy, and we include this in the associated software package. We define this as:


(2.3) $$\begin{array}{c} score=\min\left(R^{2}\right)\cdot \left(1-\frac{n_{\text{included}}}{n_{\text{total}}}\right). \end{array}$$

This provides an approximate metric representing a balance between brevity and accuracy based on the minimum $R^{2}$ achieved across dimensions (e.g. different subscales) of the target variable. By including a term that depends upon the number of items included ($1-\frac{n_{\text{included}}}{n_{\text{total}}}$), we penalize models that include a greater proportion of the original items, such that we prefer models that are accurate (based on $R^{2}$) but which include fewer items. By taking the minimum $R^{2}$ across multiple dimensions, we ensure that the resulting solution predicts scores accurately across dimensions; while we could use the mean or median, this could result in a model that appears successful but is not consistently accurate across dimensions. In general, however, the model selected will be dependent on situation-specific requirements. As such, while we provide this metric for utility, we focus in our examples on the variety of potential solutions rather than a single ‘correct’ solution.

We further sought to provide an indication of how dependent this procedure is on sample size, since in general, models trained with larger samples will make more accurate predictions. To achieve this, we repeated our analysis with different sample sizes (*n* = 50, 100, 200, 300, 400, 500). For each, we generated 1000 subsamples with replacement from our data, providing an indication of both how sample size affects accuracy and how variable this is across varying datasets. In each subsample, we tested models with every possible number of retained items and calculated the $R^{2}$ for each.

### 2.3. Pipeline overview

These steps can be assembled to produce a straightforward pipeline for estimating scores, which we summarize here for clarity:

(1) For each dimension in the data, fit a Lasso-regularized regression model predicting scores on this dimension from individual items. This should be performed in the training dataset.
(2) Take the items that are present in at least one of these models (i.e. combine all the items with a non-zero coefficient across the models for each dimension). Fit new unregularized regression models predicting the value of each dimension from only these items. This should be performed in the training dataset. This step is not required if there is only a single dimension to be predicted.
(3) Evaluate the predictive accuracy of these models on a validation dataset (e.g. using $R^{2}$). Predictions are made by participants’ responses to the included items by their weights in the model.
(4) Repeat this procedure for a number of iterations, using a different set of regularization parameter values α in each iteration. The number of iterations will depend upon the complexity of the problem; more complex problems (e.g. with more dimensions) will require more iterations.
(5) Select the model that performs best according to the desired criteria (e.g. balancing number of included items against predictive accuracy).
(6) Evaluate the performance of this chosen model in the test dataset.

Together, this provides a straightforward procedure for reducing the number of items in a given measure, ensuring that it retains the ability to accurately predict scores.

### 2.4. Implementation

We have developed a Python package which implements the FACSIMILE method, which is available online (https://github.com/the-wise-lab/FACSIMILE) [23]. This is designed to be straightforward and usable with little need for additional configuration and implements the optimization procedures described above. Documentation and examples are provided within the above repository.

Once a model is trained and selected, weights for the individual items can also be extracted easily to be used outside of this package. For example, it may be desirable to create a simple spreadsheet that can calculate predicted scores without the need for any knowledge of coding by simply multiplying the weights by the entered item scores.

### 2.5. Example data

Here, we demonstrate the FACSIMILE approach using an example dataset containing responses to a commonly used trait anxiety questionnaire with an established two-factor structure, the state trait inventory of cognitive and somatic anxiety trait version (STICSA). This dataset includes responses from 1622 participants who completed the STICSA across multiple studies. We split this dataset into a training set of 972 participants, a validation set of 325 participants and a test set of 325 participants.

### 2.6. Exploratory factor analysis

To demonstrate the ability of FACSIMILE to accurately estimate factor scores derived from exploratory factor analysis, we use the procedure to derive a two-factor solution for the STICSA, following the original description of the measure. We run this analysis in Python using the FactorAnalyzer package (https://github.com/EducationalTestingService/factor_analyzer). We perform this using maximum likelihood estimation and an oblimin rotation and determine the number of factors according to the scree plot.

## 3. Results

We demonstrate the effectiveness of our approach using an example dataset of responses to the STICSA, a trait anxiety measure with a two-factor structure [24].

### 3.1. Predicting sum scores

**Figure 1.**
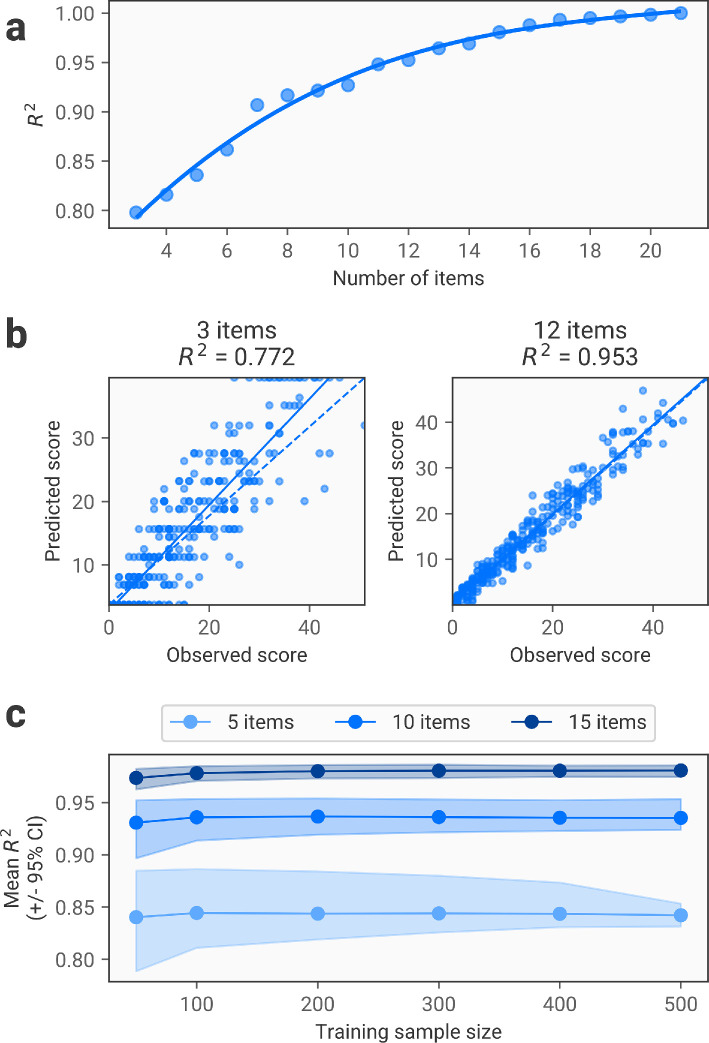
Item reduction using FACSIMILE for sum scores. (a) Relationship between the number of included items and accuracy in predicting observed sum scores, as quantified by the $R^{2}$ score. The solid line represents a cubic fit to the results. Note that there is only one point per number of items, as with a single dimension only a single combination of items will be identified for each level of regularization. (b) Scatter plots showing observed versus predicted sum scores for each participant in the test dataset. The left figure shows the results using a variant with only three items included, while the right figure shows a variant with 12 items included. The dashed line represents perfect prediction (observed = predicted), while the solid line represents a linear fit to the results. (c) Analysis of sensitivity to training sample size. The solid lines represent the mean $R^{2}$ achieved for a model of the corresponding sample size and number of retained items, while the shaded area represents the 95% confidence interval around this value.

We first use the FACSIMILE method to predict sum scores on the measure (i.e. summing the responses to every item in the scale). We run 1000 iterations of the procedure with different alpha values drawn from a beta distribution with a scaling factor of 8 (i.e. values from the distribution are multiplied by 8). The results of this procedure are shown in figure 1, which demonstrates how predictive accuracy increases as a function of the number of items included. Note that here the steps in item inclusions are relatively coarse-grained, being a short measure (22 items). Further, the optimization procedure is somewhat redundant as we are not predicting multiple dimensions; multiple iterations of the procedure with similar α values will inevitably lead to the same number of included items with the same predictive accuracy.

The results show that this procedure is effective in reducing the number of items required to adequately estimate sum scores on the measure. Even with fewer than 10 items, sum scores can be estimated with $R^{2}$ scores of over 0.9, indicating high accuracy. As described in the methods, there is no ‘optimal’ number of items, and this provides a variety of options depending on the extent to which a researcher wishes to shorten the measure. To provide an indication of the sample size required to train such a model, we repeated this procedure using varying sample sizes. This demonstrated that performance began to plateau at a relatively small sample size of around 100 (figure 1c). However, performance was in general still very high even with smaller samples; even with the smallest sample size used (50 participants), accuracy was only marginally lower than with far larger sample sizes.

### 3.2. Predicting subscale scores

We can extend the method to predict scores on subscales. The STICSA has two subscales for cognitive and somatic anxiety [24], and so we train models to predict these subscales based on a subset of the measure’s items. This follows the same procedure as above, but we evaluate different α values for the different subscales. This results in a single final set of items that can be used to predict both dimensions.

**Figure 2.**
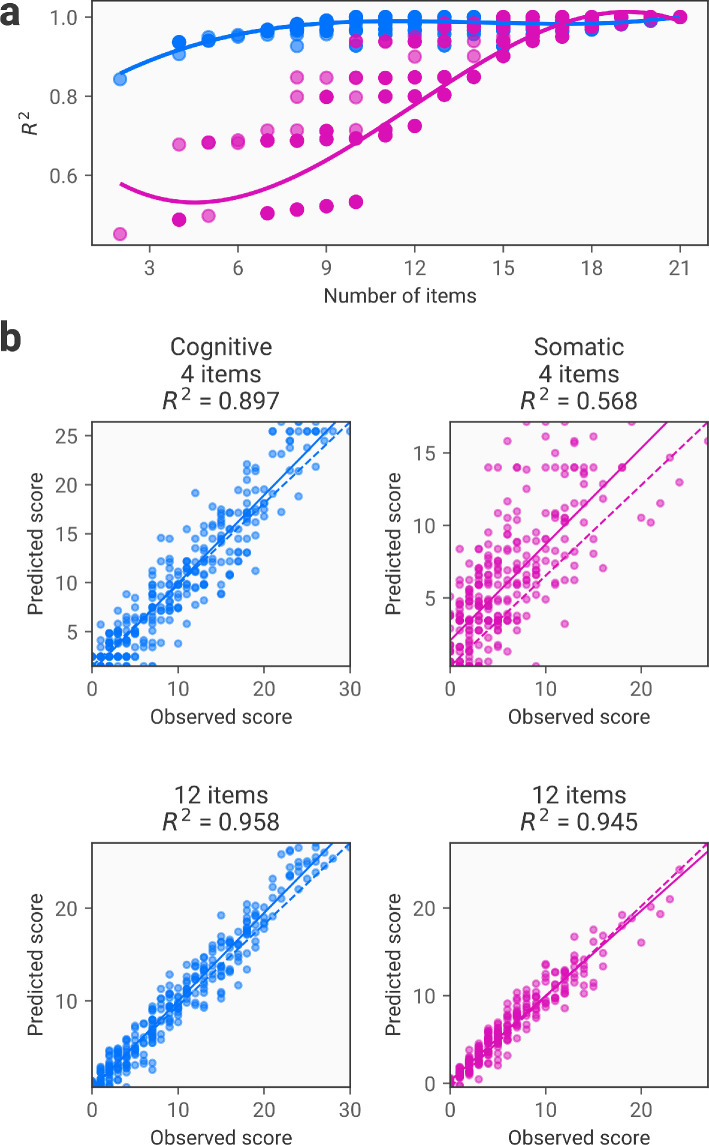
Item reduction using FACSIMILE for subscale scores. (a) Relationship between the number of included items and accuracy in predicting observed subscale scores, as quantified by the $R^{2}$ score. Here, the number of items refers to the final number of items that are used for predicting both subscales. Note that performance for a given number of items varies depending on the particular model, as different models may include different combinations of items (shown by the different dots for a given number of items) depending on the combination of α values used. (b) Scatter plots showing observed versus predicted sum scores for each participant in the test dataset. The top row shows the results using a variant with four items included, with the cognitive subscale on the left and the somatic subscale on the right. The bottom row shows a variant with 12 items included, again with the cognitive and somatic subscales on the left and right, respectively. Again, predictions are derived from a final model and item set that is used to predict both dimensions. Note that predictive accuracy may differ from (a) to (b) since these are derived from different datasets: the validation and test dataset, respectively.

As shown in figure 2, the cognitive subscale is predicted more accurately with lower item numbers than the somatic subscale. Thus, a substantially shortened measure (below around 10 items) will be able to predict scores on one subscale with satisfactory accuracy, but will perform poorly in predicting the other subscale. Nonetheless, we can derive a shortened scale with $R^{2}$ scores of over 0.9 for both subscales with as few as 10 items, representing over a 50% reduction in scale length. This procedure provides multiple candidate models for a given number of included items, as shown in figure 2a, where we often see a range of $R^{2}$ scores for the same number of included items. This results from having different combinations of items for each dimension, based on the α values to determine the strength of regularization. For example, we may have two 10 item solutions, one with 9 items from the Cognitive subscale and one with 9 items from the Somatic subscale; both of these solutions would have the same number of items but may differ substantially in their performance.

### 3.3. Predicting factor scores

The FACSIMILE method can also be applied to factors derived from exploratory factor analysis to predict individual participants’ factor scores from a reduced set of items. To demonstrate this, we perform exploratory factor analysis on the STICSA, which has an established two-factor structure [24].

**Figure 3.**
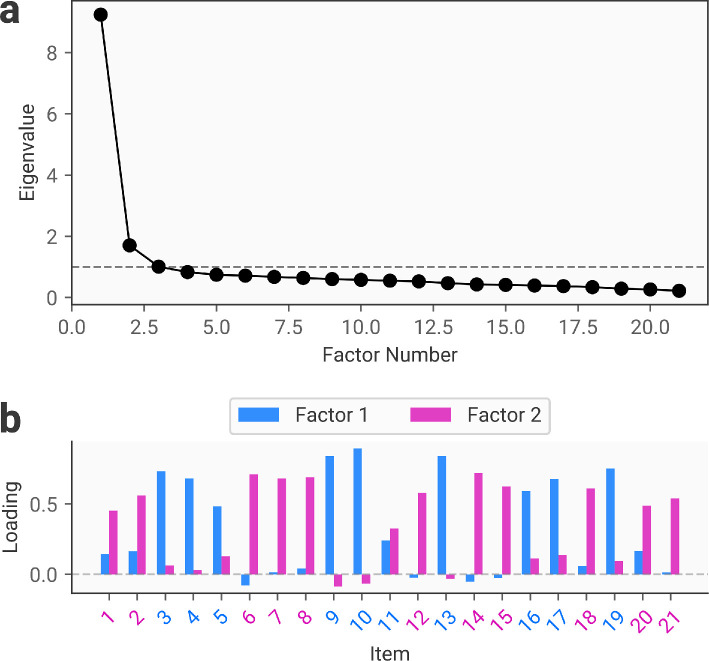
Results of the exploratory factor analysis performed on the STICSA data. (a) The scree plot, demonstrating evidence for the two-factor solution. (b) The factor loadings for each item in the measure. Factors 1 and 2 putatively correspond to cognitive and somatic factors. The item numbers are colour-coded according to their belonging to the cognitive and somatic factors in the original article, and correspondence between our solution and the original factor analysis can be observed based on the correspondence between these colours and the colour of the maximal loading for each item (i.e. blue items should have high loadings on the blue factor).

As shown in figure 3, the scree plot indicates that a 2-factor solution best describes the data. Examining the item loadings, we can observe that the two factors replicate those identified in the original article, capturing cognitive and somatic dimensions. We note that this approach could also be applied to solutions from confirmatory factor analysis based on established factor structures derived in prior research, rather than re-deriving a known factor structure using exploratory factor analysis.

**Figure 4.**
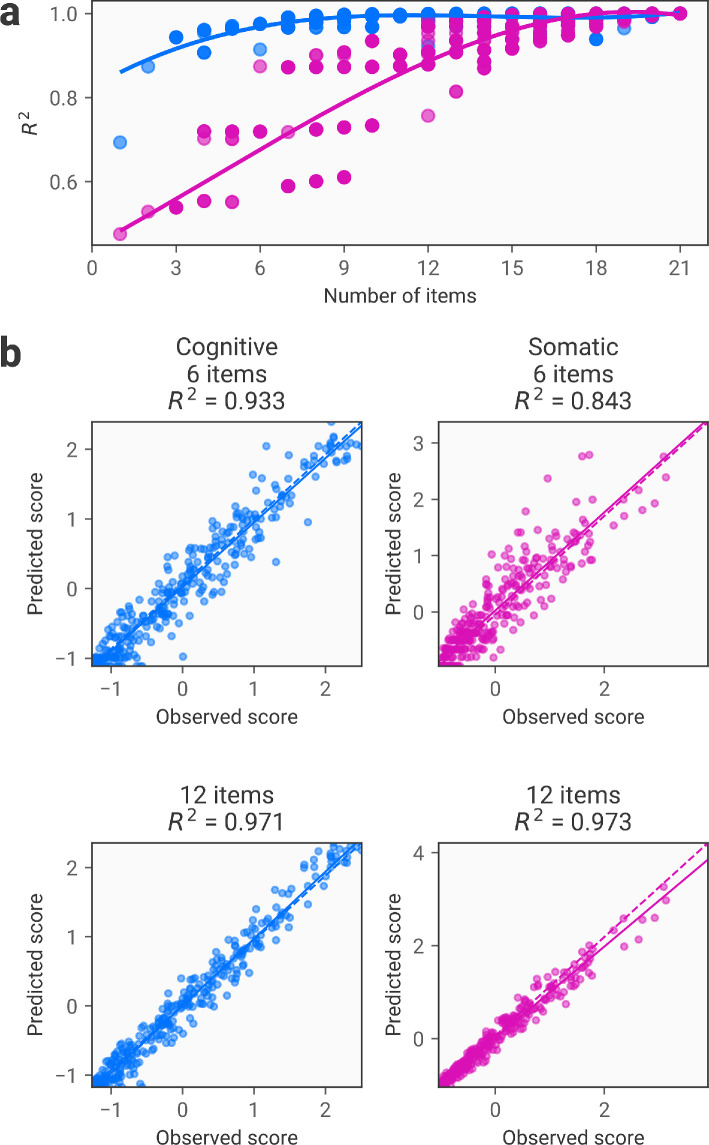
Item reduction using FACSIMILE for factor scores derived from exploratory factor analysis. (a) Relationship between the number of included items and accuracy in predicting observed factor scores, as quantified by the $R^{2}$ score. Here, the number of items refers to the final number of items that are used for predicting both subscales. As in figure 2, the results represent different potential combinations for each number of included items depending on the combination of α values used (b) Scatter plots showing observed versus predicted sum scores for each participant in the test dataset. The top row shows the results using a variant with 6 items included, with the cognitive factor on the left and the somatic factor on the right. The bottom row shows a variant with 12 items included with the cognitive and somatic factors on the left and right, respectively. Again, predictions are derived from a final model and item set that is used to predict both dimensions. Note that predictive accuracy may differ from (a) to (b) since these are derived from different datasets: the validation and test dataset, respectively.

We next apply the FACSIMILE procedure to generate a reduced set of items that can accurately predict participants’ scores on the two factors. In practice, this follows the same procedure as the above analysis predicting subscales, the only difference being that we are calculating factor scores derived from exploratory factor analysis rather than sum scores on the subscales identified through factor analysis (i.e. summing responses to the items that most strongly load on to each factor). As before, it is possible to accurately estimate participants’ factor scores using a subset of items that is approximately 50% shorter than the full measure figure 4.

## 4. Discussion

Here, we introduce the FACSIMILE method for creating short scales. This method takes a data-driven approach, selecting a subset of items that can be combined using linear weighting to accurately estimate sum scores, subscale scores or factor scores based on a reduced set of items. The method is accurate, straightforward to use, and is not subject to some of the limitations of existing methods for the creation of short scales.

Our method builds on existing approaches for the creation of short scales, such as those using item response theory [12]. Our method diverges from these approaches by using a data-driven approach based on predictive accuracy, rather than seeking to build a model of the relationship between responses to individual items and values of an underlying latent construct. We also introduce the use of linearly weighted item combinations through regression to enable more accurate predictions than using a simple sum of item responses. This method provides a straightforward approach for creating short scales that can both reduce participant burden and make data collection more economical. To further ease its use, we have developed a Python package that enables users to apply the method without the need to specify machine learning models directly.

Notably, we observed that reasonable accuracy ($R^{2}$ of 0.8 or higher) in our example dataset could be achieved with as few as three items. In settings where perfect accuracy is not essential but researchers wish to acquire an approximate estimate in a short time, this could provide for a quick and simple method of doing so. The relationship between the number of included items and accuracy appeared to be nonlinear, with the implication that a substantial number of items (approx. 50%) can be dropped from the measure while retaining high predictive accuracy ($R^{2}$ of 0.95 or higher). Furthermore, while performance improved somewhat with larger training samples, we were able to train highly accurate models even with relatively small samples of around 50 participants. The sample size required for other use cases will depend on the psychometric properties of the measure being used, however. For example, scales that are designed to produce very skewed responses when used in unselected populations (as is the case with screening measures that aim to distinguish between cases and controls) may pose a greater challenge.

While we have demonstrated the most likely uses of this method, it is highly flexible and may be of use in other situations. For example, we might imagine a situation where we have scores on multiple factors derived from exploratory factor analysis, but only wish to predict one score in a new study; a shortened measure could be developed to predict just this single factor. Alternatively, we may have factor solutions of varying complexity [25] and wish to predict scores on each of the factors simultaneously with a shortened measure. Furthermore, the inclusion of items is flexible. As an example, we may have an established set of factors derived from 10 questionnaire measures combined, and wish to estimate scores on these factors in a dataset where we have data for only five of these measures. Using the original dataset, we can simply train a model to predict the observed factor scores that only include items from these five measures, and then apply it in our new dataset.

A question we have not addressed here is the extent to which these reduced scales are able to predict external measures of interest (such as other questionnaire measures or aspects of behaviour); i.e. do predicted scores represent the same construct as the observed scores in terms of their external validity. However, we and others have successfully used earlier versions of this methodology to successfully relate shortened measures of mental health symptoms to behaviour across multiple studies [19,26–28], suggesting that the predicted scores do not differ in their relationships with external variables compared with the observed scores. More generally, while we have not tested this here, many psychometric properties of the predicted scores (e.g. test–retest reliability) should be similar to observed scores so long as the model is accurate. Future work should seek to confirm this, and verify that psychometric properties derived from short scales are consistent with those of the full scale. Finally, these predicted scores will inevitably be subject to limitations of scores derived from the full scale and will still represent indirect and somewhat noisy measures of a true underlying psychological construct.

It is important to note that our approach does have limitations and will not be viable in every setting. Predictive accuracy will depend on the number of included items and will never be perfect; in settings where perfect accuracy is essential (e.g. in clinical settings), this may not be acceptable. The method also relies on a weighted combination of items, in comparison to typical shortened measures for which sum scores can be calculated straightforwardly by summing responses. This makes the calculation of scores marginally more complex, although this is far from prohibitive and enables greater accuracy and brevity. Our method also does not provide the deeper insights into the nuances of a self-report measure that approaches such as item response theory can bring. Rather, it is a simple and effective, but blunt, tool that aims to estimate scores without any deeper understanding of how the measure is constructed. Finally, any approximate scores derived through our approach will only be as valid as the true scores derived from the full scale; an accurate prediction of scores on an invalid or unreliable measure will have lesser utility than those from a robust and well-validated measure.

## Data Availability

The Python package implementing the FACSIMILE method, along with code and data to reproduce the examples given here, is available at [29] and have been archived within a Zenodo repository [23].

## References

[1] Gibson AM, Bowling NA. 2020 The effects of questionnaire length and behavioral consequences on careless responding. Eur. J. Psychol. Assess. **36**, 410–420. (10.1027/1015-5759/a000526)

[2] Herzog AR, Bachman JG. 1981 Effects of questionnaire length on response quality. Public Opin. Q. **45**, 549–559. (10.1086/268687)

[3] McCluskey S, Topping A. 2011 Increasing response rates to lifestyle surveys: a pragmatic evidence review. Perspect. Public Health **131**, 89–94. (10.1177/1757913910389423) 21462753

[4] Rolstad S, Adler J, Rydén A. 2011 Response burden and questionnaire length: is shorter better? A review and meta-analysis. Value Health **14**, 1101–1108. (10.1016/j.jval.2011.06.003) 22152180

[5] Ziegler M, Kemper CJ, Kruyen P. 2014 Short scales – five misunderstandings and ways to overcome them. J. Individ. Differ. **35**, 185–189. (10.1027/1614-0001/a000148)

[6] Cai L, Choi K, Hansen M, Harrell L. 2016 Item response theory. Annu. Rev. Stat. Its Appl. **3**, 297–321. (10.1146/annurev-statistics-041715-033702)

[7] Edelen MO, Reeve BB. 2007 Applying item response theory (IRT) modeling to questionnaire development, evaluation, and refinement. Qual. Life Res. **16**, 5–18. (10.1007/s11136-007-9198-0) 17375372

[8] Allen VD, Weissman A, Hellwig S, MacCann C, Roberts RD. 2014 Development of the situational test of emotional understanding – brief (STEU-B) using item response theory. Personal. Individ. Differ. **65**, 3–7. (10.1016/j.paid.2014.01.051)

[9] Chiesi F, Morsanyi K, Donati MA, Primi C. 2018 Applying item response theory to develop a shortened version of the need for cognition scale. Adv. Cogn. Psychol. **14**, 75–86. (10.5709/acp-0240-z) 32337000 PMC7171511

[10] Sekely A, Taylor GJ, Bagby RM. 2018 Developing a short version of the Toronto structured interview for Alexithymia using item response theory. Psychiatry Res. **266**, 218–227. (10.1016/j.psychres.2018.03.002) 29609989

[11] Sturm A, Kuhfeld M, Kasari C, McCracken JT. 2017 Development and validation of an item response theory‐based social responsiveness scale short form. J. Child Psychol. Psychiatry **58**, 1053–1061. (10.1111/jcpp.12731) 28464350

[12] Thissen D, Steinberg L. 1986 A taxonomy of item response models. Psychometrika **51**, 567–577. (10.1007/bf02295596)

[13] Cai L, Thissen D. 2014 Modern approaches to parameter estimation in item response theory. In Handbook of item response theory modeling. Abingdon-on-Thames, UK: Routledge.

[14] Maydeu-Olivares A. 2013 Goodness-of-fit assessment of item response theory models. Meas. Interdiscip. Res. Perspect. **11**, 71–101. (10.1080/15366367.2013.831680)

[15] Reise SP, Cook KF, Moore TM. 2014 Handbook of item response theory modeling. Abingdon-on-Thames, UK: Routledge. (10.4324/9781315736013-11)

[16] Gillan CM, Kosinski M, Whelan R, Phelps EA, Daw ND. 2016 Characterizing a psychiatric symptom dimension related to deficits in goal-directed control. eLife **5**, e11305. (10.7554/elife.11305) 26928075 PMC4786435

[17] Wise T, Robinson OJ, Gillan CM. 2023 Identifying transdiagnostic mechanisms in mental health using computational factor modeling. Biol. Psychiatry **93**, 690–703. (10.1016/j.biopsych.2022.09.034) 36725393 PMC10017264

[18] Hopkins AK, Gillan C, Roiser J, Wise T, Sidarus N. 2022 Optimising the measurement of anxious-depressive, compulsivity and intrusive thought and social withdrawal transdiagnostic symptom dimensions. (10.31234/osf.io/q83sh)

[19] Wise T, Dolan RJ. 2020 Associations between aversive learning processes and transdiagnostic psychiatric symptoms in a general population sample. Nat. Commun. **11**, 4179. (10.1038/s41467-020-17977-w) 32826918 PMC7443146

[20] Argyriou A, Evgeniou T, Pontil M. 2008 Convex multi-task feature learning. Mach. Learn. **73**, 243–272. (10.1007/s10994-007-5040-8)

[21] Nie F, Huang H, Cai X, Ding C. 2010 Efficient and robust feature selection via joint \mathscrl2,1-norms minimization. In Advances in neural information processing systems. Curran associates, Inc.

[22] Bergstra J, Bengio Y. 2012 Random search for hyper-parameter optimization. J. Mach. Learn. Res. **13**, 281–305.

[23] Wise T, Sookud S. 2025 the-wise-lab/FACSIMILE: FACSIMILE v0.1.2 (v0.1.2). Zenodo. (10.5281/zenodo.14973435)

[24] Grös DF, Antony MM, Simms LJ, McCabe RE. 2007 Psychometric properties of the state-trait inventory for cognitive and somatic anxiety (STICSA): comparison to the state-trait anxiety inventory (STAI). Psychol. Assess. **19**, 369–381. (10.1037/1040-3590.19.4.369) 18085930

[25] Wise T, Sookud S, Michelini G, Mobbs d. 2024 Transdiagnostic mental health symptom dimensions predict use of flexible model-based inference in complex environments (10.31234/osf.io/f49gr) PMC12982692 41794888

[26] Donegan KR, Brown VM, Price RB, Gallagher E, Pringle A, Hanlon AK, Gillan CM. 2023 Using smartphones to optimise and scale-up the assessment of model-based planning. Commun. Psychol. **1**, 1–15. (10.1038/s44271-023-00031-y) 39242869 PMC11332031

[27] Fox CA, McDonogh A, Donegan KR, Hanlon A, Gallagher E, Rouault M, Gillan C. 2023 Reliable, rapid, and remote measurement of metacognitive bias. (10.31234/osf.io/c5abx) PMC11213917 38942811

[28] Sookud S, Martin I, Gillan C, Wise T. 2024 Impaired goal-directed planning in transdiagnostic compulsivity is explained by uncertainty about learned task structure. (10.31234/osf.io/zp6vk) 41110550

[29] Wise T. 2025 FACtor Score IteM reductIon with Lasso Estimator (FACSIMILE). GitHub. https://github.com/the-wise-lab/FACSIMILE

